# Wastewater Testing and Detection of Poliovirus Type 2 Genetically Linked to Virus Isolated from a Paralytic Polio Case — New York, March 9–October 11, 2022

**DOI:** 10.15585/mmwr.mm7144e2

**Published:** 2022-11-04

**Authors:** A. Blythe Ryerson, Daniel Lang, Mohammed A. Alazawi, Milagros Neyra, Dustin T. Hill, Kirsten St. George, Meghan Fuschino, Emily Lutterloh, Bryon Backenson, Samuel Rulli, Patricia Schnabel Ruppert, Jacqueline Lawler, Nancy McGraw, Andrew Knecht, Irina Gelman, Jane R. Zucker, Enoma Omoregie, Sarah Kidd, David E. Sugerman, Jaume Jorba, Nancy Gerloff, Terry Fei Fan Ng, Adriana Lopez, Nina B. Masters, Jessica Leung, Cara C. Burns, Janell Routh, Stephanie R. Bialek, M. Steven Oberste, Eli S. Rosenberg, 2022 U.S. Poliovirus Response Team, 2022 U.S. Poliovirus Response Team, Bridget J. Anderson, Noelle Anderson, Jed A. Augustine, Megan Baldwin, Kelly Barrett, Ursula Bauer, Andrew Beck, Hanen Belgasmi, Lydia J. Bennett, Achal Bhatt, Debra Blog, Heather Boss, Isaac Ravi Brenner, Barrett Brister, Travis Wayne Brown, Tavora Buchman, James Bullows, Kara Connelly, Blaise Cassano, Christina J. Castro, Cynthia Cirillo, George Edward Cone, Janine Cory, Amina Dasin, Adina de Coteau, Anny DeSimone, Francoise Chauvin, Cynthia Dixey, Kathleen Dooling, Shani Doss, Christopher Duggar, Christopher N. Dunham, Delia Easton, Christina Egan, Brian D. Emery, Randall English, Nicola Faraci, Hannah Fast, G. Stephanie Feumba, Nancy Fischer, Stephen Flores, Ann D. Frolov, Halle Getachew, Brittany Gianetti, Alejandro Godinez, Todd Gray, William Gregg, Christina Gulotta, Sarah Hamid, Tiffany Hammette, Rafael Harpaz, Lia Haynes Smith, Brianna Hanson, Elizabeth Henderson, Eugene Heslin, Alexandra Hess, Dina Hoefer, Jonathan Hoffman, Lyndsey Hoyt, Scott Hughes, Anna Rose Hutcheson, Tabassum Insaf, Christopher Ionta, Stacey Jeffries Miles, Anita Kambhampati, Haley R. Kappus-Kron, Genevieve N. Keys, Michael Kharfen, Gimin Kim, Jenna Knox, Stephanie Kovacs, Julie Krauchuk, Elisabeth R. Krow-Lucal, Daryl Lamson, Jennifer Laplante, David A. Larsen, Ruth Link-Gelles, Hongmei Liu, James Lueken, Kevin Ma, Rachel L. Marine, Karen A. Mason, James McDonald, Kathleen McDonough, Kevin McKay, Eva McLanahan, Eric Medina, Haillie Meek, Gul Mehnaz Mustafa, Megan Meldrum, Elizabeth Mello, Jeffrey W. Mercante, Mandar Mhatre, Susan Miller, Natalie Migliore, Neida K. Mita-Mendoza, Amruta Moghe, Nehalraza Momin, Tanner Morales, E. Joe Moran, Grace Nabakooza, Dana Neigel, Simon Ogbamikael, Jason O’Mara, Stephanie Ostrowski, Manisha Patel, Prabasaj Paul, Atefeh Paziraei, Georgina Peacock, Lauren Pearson, Jonatha Plitnick, Alicia Pointer, Michael Popowich, Chitra Punjabi, Rama Ramani, Shailla J. Raymond, Lindsey Rickerman, Erik Rist, Angela C. Robertson, Shannon L. Rogers, Jennifer B. Rosen, Cecelia Sanders, Jeanne Santoli, Leanna Sayyad, Lynsey Schoultz, Matthew Shudt, Justin Smith, Theresa L. Smith, Maria Souto, Ashleigh Staine, Shannon Stokley, Hong Sun, Andrew J. Terranella, Ashley Tippins, Farrell Tobolowsky, Megan Wallace, Steve Wassilak, Amanda Wolfe, Eileen Yee

**Affiliations:** ^1^2022 CDC Domestic Poliovirus Emergency Response Team; ^2^New York State Department of Health; ^3^Department of Public Health, Syracuse University, Syracuse, New York; ^4^Department of Biomedical Science, State University of New York at Albany, Albany, New York; ^5^Rockland County Department of Health, Pomona, New York; ^6^Orange County Department of Health, Goshen, New York; ^7^Sullivan County Department of Public Health, Liberty, New York; ^8^Nassau County Department of Health, Mineola, New York; ^9^New York City Department of Health and Mental Hygiene, New York, New York; ^10^Epidemic Intelligence Service, CDC; ^11^Department of Epidemiology and Biostatistics, State University of New York at Albany, Albany, New York.; New York State Department of Health; CDC; CDC; New York State Department of Health; New York State Department of Health; New York State Department of Health; CDC; CDC; New York State Department of Health; CDC; CDC; Orange County Department of Health; New York State Department of Health; CDC; CDC; Nassau County Department of Health; CDC; New York State Department of Health; Rockland County Department of Health; CDC; Nassau County Department of Health; CDC; CDC; CDC; CDC; Nassau County Department of Health; New York City Department of Health and Mental Hygiene; CDC; CDC; CDC; CDC; Syracuse University; New York State Department of Health; Wadsworth Center, New York State Department of Health; CDC; CDC; Wadsworth Center, New York State Department of Health; CDC; Wadsworth Center, New York State Department of Health; Nassau County Department of Health; CDC; CDC; CDC; CDC; New York State Department of Health; Wadsworth Center, New York State Department of Health; CDC; New York State Department of Health; CDC; CDC; CDC; CDC; New York State Department of Health; CDC; New York State Department of Health; CDC; New York State Department of Health; New York City Department of Health and Mental Hygiene; New York State Department of Health; New York City Department of Health and Mental Hygiene; CDC; New York State Department of Health; CDC; CDC; CDC; New York State Department of Health; CDC; New York State Department of Health; CDC; Sullivan County Department of Public Health; CDC; Rockland County Department of Health; CDC; Wadsworth Center, New York State Department of Health; Wadsworth Center, New York State Department of Health; Syracuse University; CDC; CDC; CDC; CDC; CDC; CDC; New York State Department of Health; Wadsworth Center, New York State Department of Health; Rockland County Department of Health; CDC; Rockland County Department of Health; CDC; CDC; New York State Department of Health; Rockland County Department of Health; CDC; Rockland County Department of Health; New York State Department of Health; Wadsworth Center, New York State Department of Health; New York State Department of Health; Wadsworth Center, New York State Department of Health; CDC; Rockland County Department of Health; New York State Department of Health; CDC; New York State Department of Health; Wadsworth Center, New York State Department of Health; Rockland County Department of Health; New York State Department of Health; CDC; CDC; CDC; CDC; CDC; Wadsworth Center, New York State Department of Health; Orange County Department of Health; Wadsworth Center, New York State Department of Health; Rockland County Department of Health; Wadsworth Center, New York State Department of Health; New York State Department of Health; Wadsworth Center, New York State Department of Health; Wadsworth Center, New York State Department of Health; CDC; CDC; New York City Department of Health and Mental Hygiene; CDC; CDC; CDC; Wadsworth Center, New York State Department of Health; Wadsworth Center, New York State Department of Health; CDC; CDC; Rockland County Department of Health; CDC; CDC; CDC; CDC; CDC; CDC; CDC; CDC; Sullivan County Department of Public Health; CDC

In July 2022, a case of paralytic poliomyelitis resulting from infection with vaccine-derived poliovirus (VDPV) type 2 (VDPV2)^§^ was confirmed in an unvaccinated adult resident of Rockland County, New York (*1*). As of August 10, 2022, poliovirus type 2 (PV2)^¶^ genetically linked to this VDPV2 had been detected in wastewater** in Rockland County and neighboring Orange County (*1*). This report describes the results of additional poliovirus testing of wastewater samples collected during March 9–October 11, 2022, and tested as of October 20, 2022, from 48 sewersheds (the community area served by a wastewater collection system) serving parts of Rockland County and 12 surrounding counties. Among 1,076 wastewater samples collected, 89 (8.3%) from 10 sewersheds tested positive for PV2. As part of a broad epidemiologic investigation, wastewater testing can provide information about where poliovirus might be circulating in a community in which a paralytic case has been identified; however, the most important public health actions for preventing paralytic poliomyelitis in the United States remain ongoing case detection through national acute flaccid myelitis (AFM) surveillance^††^ and improving vaccination coverage in undervaccinated communities. Although most persons in the United States are sufficiently immunized, unvaccinated or undervaccinated persons living or working in Kings, Orange, Queens, Rockland, or Sullivan counties, New York should complete the polio vaccination series as soon as possible.

High rates of poliovirus vaccination coverage (*2*) resulted in the elimination of paralytic polio caused by wild-type poliovirus in the United States in 1979.^§§^ Only inactivated polio vaccine (IPV) has been used in the United States since 2000; 3 doses of IPV confer 99%–100% protection from paralytic poliomyelitis (*3*). Some countries still use oral poliovirus vaccine (OPV); advantages to this approach include low cost, ease of use, and high efficacy in stopping outbreaks. However, in rare cases, the live attenuated virus in OPV can regain neurovirulence, circulate in underimmunized populations, and cause paralytic disease. A previous report confirmed that paralysis of the Rockland County patient resulted from infection with VDPV2, and that related viruses had been detected in wastewater collected from Orange and Rockland counties (*1*). Since then, the New York State Department of Health (NYSDOH); Nassau, Orange, Putnam, Rockland, Suffolk, Sullivan, Ulster, and Westchester counties’ health departments; New York City Department of Health and Mental Hygiene (NYC DOHMH); New York City Department of Environmental Protection; and CDC have expanded poliovirus wastewater testing as part of an emergency response. This report summarizes findings from the more extensive wastewater testing conducted in the New York metropolitan area as part of investigations to understand the extent of poliovirus circulation and to direct polio vaccination efforts.

Wastewater samples, including some originally collected for SARS-CoV-2 surveillance, were collected from a subset of sewersheds during March 9–October 11, 2022. Samples were collected approximately once or twice weekly from each site. Wastewater samples were processed using either ultracentrifugation or polyethylene glycol precipitation followed by nucleic acid extraction. The extracts were forwarded to the Wadsworth Center (part of NYSDOH) or the New York City Public Health Laboratory (part of NYC DOHMH) where they were packaged and shipped to CDC. At CDC, total nucleic acids were screened for the presence of PV2 using the pan-poliovirus real-time reverse transcription–polymerase chain reaction (RT-PCR) assay, and positive samples were sequenced (*4*,*5*).

To investigate the number of indeterminate^¶¶^ results from some of the New York City samples from large sewersheds (those servicing more than 700,000 residents), NYC DOHMH collected additional larger volume (500 mL) wastewater samples from two sewersheds on August 11, one receiving wastewater from parts of New York County, and another with combined wastewater from parts of Kings, New York, and Queens counties (two distinct upstream sub-sewersheds*** were sampled, one feeding only from the New York County area and another feeding from Kings and Queens counties combined). CDC then concentrated virus from the samples using the filtration and elution method, followed by inoculation of concentrates onto susceptible cell lines to isolate polioviruses (*6*). Cultures exhibiting viral cytopathic effect were screened by real-time RT-PCR to identify polioviruses (*4*) and sequenced as described. Data presented are from samples collected during March 9–October 11, 2022, and testing conducted through October 20, 2022.

The 48 sewersheds tested serve parts of 13 counties in New York, with a total population of approximately 11,413,000 persons (*7*). A total of 1,076 wastewater samples were collected during March 9–October 11, 2022. Among these, 89 (8.3%) samples from 10 sewersheds tested positive for PV2. Of the 82 PV2-positive samples in the state of New York (outside of New York City), 81 (98.8%) sequences from six sewersheds in Nassau, Orange, Rockland, and Sullivan counties were linked to the virus isolated from the Rockland County patient, and the sequencing results for one sample were not adequate to determine whether it was linked to the virus isolated from the patient (Table) (Figure 1) (Figure 2). Of the seven PV2-positive samples in New York City, only one, from a sub-sewershed receiving wastewater from parts of Kings and Queens counties, was linked to the virus isolated from the patient; this sample was from one of the larger-volume samples. The other six PV2-positive New York City samples included one from Kings County that was not genetically linked to the virus isolated from the patient, and five from three different sewersheds serving parts of Kings, New York, and Richmond counties that were inadequate for sequencing. PV2-positive samples genetically linked to the virus isolated from the patient were collected on more than one occasion in Orange (June 13–October 6), Rockland (May 23–October 4), and Sullivan (July 21–October 5) counties. Only a single sample each from Nassau County on August 18 and the sub-sewershed serving parts of Kings and Queens counties on August 11 tested positive for a PV2 linked to virus isolated from the patient.

**TABLE:** Wastewater test results for poliovirus, by county — 13 counties, New York and New York City, March 9–October 11, 2022

County	No. of sampling sites*	Estimated % of county population covered by sewershed	Dates samples collected	No. of sites with any PV2-positive sample	Total no. of samples tested	No. of indeterminate samples^†^	No. of PV2-positive samples				No. of negative samples
							Total	Genetic linkage to Rockland County patient^§^			
								Unknown^¶^	No	Yes	
Nassau	4	84.6	Mar 9–Oct 6	1	**87**	2	**1**	0	0	1	84
NYC–Bronx	1	52.2	Jul 5–Oct 11	0	**26**	1	**0**	0	0	0	25
NYC–Kings	4	76.1	May 31–Oct 11	2	129**	4	**2**	1	1	0	121
NYC–New York	1	38.7	Jul 5–Oct 11	0	**26**	0	**0**	0	0	0	26
NYC–Queens	4	91.4	May 31–Oct 11	0	**112**	0	**0**	0	0	0	112
NYC–Bronx and New York^††^	1	46.2, 28.9	July 5–Oct 11	0	**26**	0	**0**	0	0	0	26
NYC–Kings, New York, and Queens^§§^	1	22.4, 31.9, 5.9	May 31–Oct 11	1	**36**	8	**4**	3	0	1^¶¶^	24
NYC–Richmond	2	96.2	May 31–Oct 11	1	**68**	0	**1**	1	0	0	67
Orange	8	45.9	Mar 9–Oct 6	1	**284**	4	**25**	1	0	24	255
Putnam	1	4.6	Mar 16–Oct 5	0	**20**	0	**0**	0	0	0	20
Rockland	6	96.1	Mar 9–Oct 6	2	**126**	2	**43**	0	0	43	81
Suffolk	3	19.1	Aug 15–Oct 4	0	**14**	0	**0**	0	0	0	14
Sullivan	3	20.5	Jul 21–Oct 6	2	**21**	0	**13**	0	0	13	8
Ulster	2	20.4	Aug 31–Oct 6	0	**18**	0	**0**	0	0	0	18
Westchester	7	83.6	Aug 28–Oct 6	0	**83**	0	**0**	0	0	0	83
**Total**	**48**	**82.9**	**Mar 9–Oct 11**	**10**	1,076**	**21**	**89**	**6**	**1**	**82**	**964**

**Abbreviations:** NYC = New York City; PV2 = poliovirus type 2.

* Sampling sites are sewersheds defined as the community area served by a wastewater collection system.

^†^ Indeterminate results include those from samples that tested positive using real-time reverse transcription polymerase chain reaction, but not enough viral material was available to complete sequencing.

^§^ In July 2022, paralytic poliomyelitis resulting from infection with vaccine-derived PV2 was confirmed in an unvaccinated adult resident of Rockland County, New York.

^¶^ Sequencing insufficient to determine relation to Rockland County patient.

** Totals include two samples from Kings County that were pending sequencing results as of October 20, 2022.

^††^ Sewershed includes portions of Bronx and New York counties.

^§§^ Sewershed includes portions of Kings, New York, and Queens counties.

^¶¶^ Large-volume sample collected from the sub-sewershed serving parts of Kings and Queens counties tested positive, but the sub-sewershed serving New York County tested negative.

**FIGURE 1:**
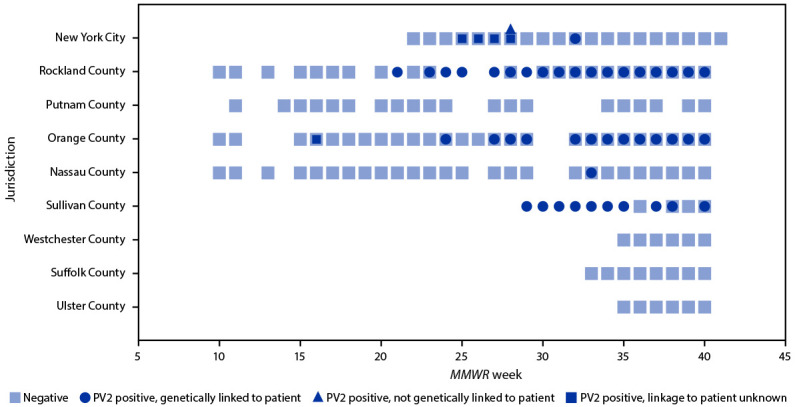
Wastewater* polio test results,^†^ by jurisdiction^§^ (N = 1,053) — 13 counties in New York and New York City, March 9–October 11, 2022 **Abbreviation:** PV2 = poliovirus type 2. * Sampling sites are sewersheds defined as the community area served by a wastewater collection system. ^†^ Testing was conducted to determine if a sample was negative or positive for PV2, and if positive for PV2, whether the PV2 was genetically linked to an unvaccinated paralytic poliomyelitis patient from Rockland County, New York identified in July 2022. Some samples had sequencing insufficient to determine relation to the Rockland County patient (i.e., linkage to patient unknown). Indeterminate results are excluded from this figure. Indeterminate results include those from samples that tested positive using real-time reverse transcription polymerase chain reaction, but not enough viral material was available to complete sequencing. Specimens pending sequencing results are also excluded. ^§^ Number of samples in each jurisdiction include New York City (408) and the following New York counties: Rockland (124), Putnam (20), Orange (280), Nassau (85), Sullivan (21), Westchester (83), Suffolk (14), and Ulster (18).

**FIGURE 2:**
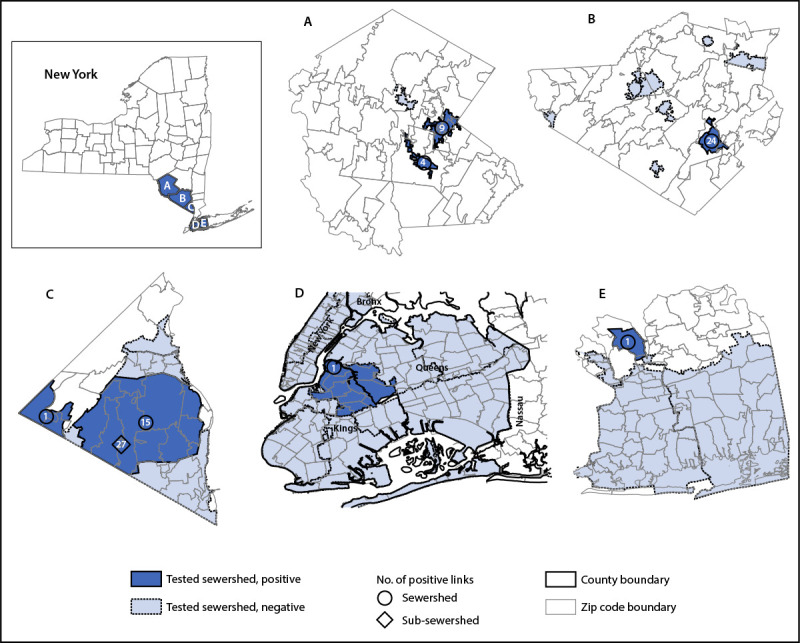
Sewersheds* with detections of poliovirus type 2 genetically linked to the virus isolated from a paralytic polio patient^†^ — Sullivan (A), Orange (B), Rockland (C), Kings and Queens (D),^§^ and Nassau (E) counties, New York, March 9–October 11, 2022 **Abbreviation:** PV2 = poliovirus type 2. * Sampling sites are sewersheds defined as the community area served by a wastewater collection system. Sub-sewersheds are upstream sampling locations within a larger sewershed. ^†^ In July 2022, paralytic poliomyelitis resulting from infection with vaccine-derived PV2 was confirmed in an unvaccinated adult resident of Rockland County, New York. ^§^ A single large-volume sample from a sub-sewershed serving parts of Kings and Queens counties tested positive for the PV2 genetically linked to the virus isolated from the patient.

In addition to wastewater testing for poliovirus in New York, a multifaceted public health response is underway that includes efforts to enhance case detection and increase vaccination access and demand. Efforts to improve case detection include testing of persons with nonparalytic, nonspecific viral symptoms consistent with poliovirus infection^†††^ and review of syndromic surveillance databases. Strategies to increase vaccination include communication campaigns, community engagement, vaccination clinics, and outreach to providers and patients, focused on communities with the lowest IPV coverage. On August 12, NYSDOH and NYC DOHMH issued a press release and health alert to guide the public and the health care community about the importance of polio vaccination, emphasizing the imperative to protect unvaccinated and undervaccinated children through vaccination.^§§§^ On September 9, New York declared a state of emergency,^¶¶¶^ which allowed additional health professionals (including certain emergency medical service providers, midwives, and pharmacists) to administer poliovirus vaccine in the state.

## Discussion

Wastewater testing during March 9–October 11 has detected PV2 genetically linked to the virus isolated from the Rockland County patient in six of 13 New York counties where wastewater was tested. One county (Nassau) had only a single detection, and therefore was not considered to have evidence of a transmission event. Three counties (Orange, Rockland, and Sullivan) had repeated detections over the course of months in one or more sewersheds, suggesting some level of community transmission in these areas. Only a single large-volume wastewater sample collected on August 11 from Kings and Queens counties in New York City tested positive for a PV2 genetically linked to virus isolated from the patient. However, this finding, coupled with the repeated PV2-positive results from the lower volume samples collected from the broader sewershed catchment areas serving parts of Kings, New York, and Queens counties during June 5–September 6 for which sequencing was not possible, suggests that PV2 could be circulating in Kings and Queens counties as well.

Wastewater testing in conjunction with high-quality AFM surveillance, has helped clarify the scope of the polio outbreak in New York, which indicates community transmission in a five-county area near the only identified symptomatic patient. Some researchers and public health agencies have had interest in expanding wastewater testing for poliovirus beyond the current outbreak area; however, additional effort is needed to understand the limitations and implications of wastewater testing for poliovirus outside the context of a localized emergency response and epidemiologic investigation of a confirmed polio case. The impact of sewershed system design and size on result interpretation needs further characterization. According to the World Health Organization’s guidelines for environmental surveillance of poliovirus circulation,**** sampling sites chosen for testing should represent selected populations at high risk with a source population of 300,000 or fewer persons. Many sewersheds in the United States, including many in New York and New York City have catchments that exceed this number by a factor of five, which could affect reliability or interpretability of results and limit the ability to effectively target interventions. Although sampling upstream sub-sewersheds can sometimes be possible, this activity might not always be feasible to do regularly because of resource and logistical constraints. In addition, monitoring the progress of polio eradication in a population with high IPV coverage is complicated by use of OPV for routine vaccination and outbreak response in other international settings. The live OPV strain can persist in stool for several weeks after vaccination, and detection of these viruses in wastewater does not have the same public health implication as does detection of a VDPV. In addition, standardized methods of testing and virus characterization need to be established if wastewater testing is to become more widespread, because reliable sequencing and careful interpretation are needed to characterize a finding in wastewater as either an OPV strain or a VDPV. Lastly, and most importantly, the public health objectives for wastewater testing for poliovirus should be defined before its application and before the public health response is scaled up beyond the currently implicated communities at risk in New York. Identifying geographies with connections to the patient’s community and persistently low polio vaccination coverage can, even in the absence of wastewater testing, help target vaccination efforts. However, these areas at risk for paralytic polio and poliovirus circulation might be considered for wastewater testing to prioritize or enhance vaccination efforts in the event of poliovirus detections.

The findings in this report are subject to at least five limitations. First, even if only a small number of persons are excreting poliovirus into a given sewershed, virus mixtures in a sample can be difficult to resolve. High-quality sequences are needed to characterize the virus and confirm linkages between viruses. Because the total number of nucleotide differences is small, a single nucleotide change can be critical in confirming a linkage between viruses. Second, defecation by infected persons in counties other than their home county in New York (e.g., where they work or visit, or through which they travel) could result in wastewater detection; hence, isolated detections do not confirm community circulation. Third, wastewater testing does not provide information about communities and facilities that are not served by municipal sewer systems; neither was every sewershed in each county sampled. Fourth, test results indicate detection or nondetection of poliovirus but cannot provide quantitative estimates of the number of persons infected. Finally, negative test results cannot guarantee that a community is free from poliovirus but can be assessed in conjunction with other surveillance approaches.

At least five New York counties had evidence of a sustained period of community transmission of poliovirus in 2022. Unvaccinated and undervaccinated persons in these areas are at risk for infection and paralytic disease. A robust national AFM surveillance system must be maintained with reporting of any suspected case of AFM to the appropriate public health authorities and collection of stool samples from any person with a suspected case. All U.S. children should receive IPV in accordance with the routine childhood immunization schedule (*8*). Most adults in the United States were vaccinated as children and are therefore likely to be protected from paralytic polio; however, any unvaccinated or undervaccinated adult or child living or working in Kings, Orange, Queens, Rockland, or Sullivan counties, New York should complete the IPV series now (*9*).

**Summary**

**What is already known about this topic?**

In July 2022, a case of paralytic poliomyelitis was confirmed in an unvaccinated adult Rockland County, New York resident; environmental sampling found evidence of poliovirus transmission.

**What is added by this report?**

Wastewater testing has identified circulating polioviruses genetically related to virus isolated from the Rockland County patient in at least five New York counties.

**What are the implications for public health practice?**

Public health efforts to prevent polio should focus on improving coverage with inactivated polio vaccine. Although most persons in the United States are sufficiently immunized, unvaccinated or undervaccinated persons living or working in Kings, Orange, Queens, Rockland, or Sullivan counties, New York should complete the polio vaccination series to prevent additional paralytic cases and curtail transmission.

## Footnotes

* These authors contributed equally to this report.

† These senior authors contributed equally to this report.

§ A VDPV is a strain related to the attenuated live poliovirus contained in OPV. VDPV2s are OPV virus strains that are >0.6% divergent (or at least six nucleotide changes) from the OPV2 strain in the complete VP1 genomic region. https://polioeradication.org/wp-content/uploads/2016/09/Reporting-and-Classification-of-VDPVs_Aug2016_EN.pdf

¶ The term PV2, referring to all serotype 2 polioviruses, is used throughout the report to indicate either a confirmed VDPV2 or a type 2 Sabin-like virus that is genetically related to the Rockland County patient. A Sabin-like poliovirus is a poliovirus that is related to one of the Sabin vaccine strains and whose nucleotide sequence in the genome region encoding the VP1 capsid protein differs from the related Sabin strain by 0–5 nucleotides for type 2 or by 0–9 nucleotides, for types 1 and 3.

** Wastewater, also referred to as sewage, includes water from household or building use (e.g., toilets, showers, and sinks) that can contain human fecal waste and water from nonhousehold sources (e.g., rain and industrial use); it does not include open drains or potable water. https://www.cdc.gov/healthywater/surveillance/wastewater-surveillance/wastewater-surveillance.html#how-wastewater-surveillance-works

††
https://www.cdc.gov/acute-flaccid-myelitis/index.html

§§ Since 1979, no cases of polio caused by wild poliovirus have originated in the United States. https://www.cdc.gov/polio/what-is-polio/polio-us.html

¶¶ Indeterminate results include those from samples that tested positive using real-time RT-PCR, but not enough viral material was available to complete sequencing.

*** Sub-sewersheds are upstream sampling locations within a larger sewershed.

†††
https://health.ny.gov/diseases/communicable/polio/docs/2022-09-28_health_advisory.pdf

§§§
https://www1.nyc.gov/site/doh/about/press/pr2022/nysdoh-and-nycdohm-wastewater-monitoring-finds-polio-urge-to-get-vaccinated.page

¶¶¶
https://health.ny.gov/press/releases/2022/2022-09-09_polio_immunization.htm

****
https://polioeradication.org/wp-content/uploads/2016/07/WHO_V-B_03.03_eng.pdf
